# Eligibility rates and representativeness of the General Medical Services scheme population in Ireland 2017-2021: A methodological report

**DOI:** 10.12688/hrbopenres.13622.1

**Published:** 2022-10-20

**Authors:** Molly Mattsson, Michelle Flood, Emma Wallace, Fiona Boland, Frank Moriarty

**Affiliations:** 1 Royal College of Surgeons in Ireland, Dublin, Ireland; 2 University College Cork, Cork, Ireland

**Keywords:** health service executive, eligibility rates, general medical services scheme, local health office

## Abstract

**Background:** In Ireland, the means tested General Medical Services (GMS) scheme provides access to a range of healthcare services at no or low cost to approximately one third of the population. Individuals eligible for the GMS scheme are often a focus of research, as a population that account for a large proportion of healthcare services use. The aim of this study is to describe the eligibility rates and representativeness of the GMS scheme population over time, with respect to age group, sex, and geographical area in Ireland.

**Methods:** Population data was obtained from the Central Statistics Office (CSO), using 2016 Census figures and projected population figures for 2017-2021. GMS eligibility figures for 2016-2021 were obtained from the HSE Primary Care Reimbursement Service (PCRS). GMS eligibility rates and relative rates of eligibility were calculated for 2016-2021 by age group and sex. Additionally, 2016 eligibility rates were calculated by geographical area.

**Results:** The crude eligibility rate decreased from 36.4% in 2016 to 31.2% in 2020, with a slight increase to 31.6% in 2021. In the 75+ years age group, 78.2% of the total population were eligible for the GMS scheme in 2021. The age group with the lowest rate of eligible individuals was the 25-34 age group, with 19.5% eligible in 2021. The eligibility rate was higher among females compared to males throughout the study period. The highest eligibility rate was seen in Donegal, with a crude rate of 52.8%. Dublin had the lowest rate, with a crude rate of 29.3%.

**Conclusions: **GMS eligibility varies greatly depending on age, sex, and geographical area, and decreased between 2016 and 2021. This study uses the most up-to-date data available to provide age group, sex and area-based figures for GMS eligibility which may inform planning and conduct of research focusing on GMS-eligible individuals.

## Introduction

Ireland currently has a mixed public-private system of healthcare. Entitlement to publicly-funded healthcare services is largely via the General Medical Services (GMS or “medical card”) scheme ^1^. This scheme provides access to a range of healthcare services at no or low cost to approximately one third of the population ^2^. This includes unlimited visits to general practitioners (GP) and other community services, emergency department visits, and hospital inpatient care, free to patients at the point of access. It also includes dispensing of a wide range of prescribed medications which are reimbursed to pharmacies by the Irish health service, where a small prescription charge applies to patients per item dispensed (currently €1.50 or €1 for those aged 70 years and over), with a monthly household cap (€15 or €10 for over 70s).

Entitlement to services under the GMS scheme is mainly based on income, with lower income thresholds for older adults resulting in higher eligibility in this group. The income thresholds have varied over time, based on government budgetary decisions. In a small proportion of cases, eligibility is granted on a discretionary basis if there is evidence of high medical expenses. In 2021, approximately 11% of all medical cards were discretionary ^3^.

Individuals eligible for the GMS scheme are often a focus of research, as a population that account for a large proportion of healthcare services use ^4^. In addition, the management of the GMS scheme results in the generation of administrative data, which represent an important resource for health research ^5^, and has been linked with other research data to facilitate pharmacoepidemiology studies ^6– 8^. An important consideration in any research focussing on GMS scheme eligible individuals as the study population is their representativeness and the potential for selection bias. Therefore, the aim of this study is to describe the eligibility rates and representativeness of the GMS scheme population over time, with respect to age group, sex, and geographical area in Ireland.

## Methods

This is a repeated cross-sectional study.

### Data sources

Population data for 2016–2021 were obtained from the Central Statistics Office (CSO) via Ireland’s Open Data Portal ^9^. The census is a legally mandatory count of the population and is completed by each household every five years. For 2016, census data from April of the same year was used, as this was when the census was last recorded. For 2017–2021, estimated population figures for April each year based on Census 2016 projections were obtained. Data included single year of age and sex for the estimated years, while Census 2016 data also included information at county level. The CSO does not provide population estimates by county, age, and sex for non-Census years. Age groups were created to match those reported for the GMS eligibility figures (0–4, 5–11, 12–15, 16–24, 25–34, 35–44, 45–54, 55–64, 65–69, 70–74, and 75+ years).

GMS eligibility figures for 2017–2021 were obtained from the Health Service Executive (HSE) Primary Care Reimbursement Service (PCRS) via the PCRS’s Reporting and Open Data platform, while 2016 figures were requested directly from the PCRS. Aggregate figures for number of GMS-eligible individuals by age group and sex were extracted for April 2016–2021 to correspond with Census population figures. For April 2016, information at HSE Local Health Office (LHO) level (i.e. geographical areas for the administration of healthcare entitlements) was also obtained. As LHOs do not correspond directly to counties, broader areas combining counties and/or LHOs were created to provide equivalence for analysis purposes (see Table 1).

**Table 1.** Local health offices (LHOs), Central Statistics Office (CSO) areas, and areas used for analysis.

Local Health Office (PCRS)	County and city (CSO)	New area
Cavan/Monaghan	Cavan	Cavan/Monaghan/ Sligo/Leitrim
	Monaghan	
Sligo/Leitrim/West Cavan	Sligo	
	Leitrim	
Donegal	Donegal	Donegal
Galway	Galway City	Galway
	Galway County	
Mayo	Mayo	Mayo
Roscommon	Roscommon	Roscommon
Clare	Clare	Clare
Limerick	Limerick	Limerick/Tipperary
North Tipperary/ East Limerick	Tipperary	
South Tipperary		
Kerry	Kerry	Kerry
North Cork	Cork City	Cork
North Lee	Cork County	
South Lee		
West Cork		
Carlow/Kilkenny	Carlow	Carlow/Kilkenny
	Kilkenny	
Waterford	Waterford	Waterford
Wexford	Wexford	Wexford
Dublin South East	Dublin City	Dublin
Dún Laoghaire	Dún Laoghaire- Rathdown	
Dublin South City	Fingal	
Dublin South West	South Dublin	
Dublin West		
Dublin North		
Dublin North Central		
Dublin North West		
Wicklow	Wicklow	Kildare/Wicklow
Kildare/West Wicklow	Kildare	
Laois/Offaly	Laois	Laois/Offaly
	Offaly	
Longford/ Westmeath	Longford	Longford/ Westmeath
	Westmeath	
Louth	Louth	Louth
Meath	Meath	Meath

### Analysis

The characteristics of the GMS population per year were summarised. Crude eligibility rates were calculated for 2016–2021 by age group and sex, and demographic group (children, 0–15 years; adults, 16–64 years; and older adults, 65+ years), along with the relative rate of eligibility (how much more or less likely a member of a particular age or sex group was to be eligible compared to the general population). The crude eligibility rate per year was calculated, and the yearly rates were also directly standardised to the 2016 population based on age group and sex.

For the area-level analysis, crude eligibility rates were calculated using Census 2016 data and April 2016 eligibility data and directly standardised to national population based on age group and sex. Standardised eligibility rates were plotted on a map by area. Analyses were conducted using Stata version 17 ^10^, and figures were generated in RStudio using the *tmap* and *ggplot2* packages ^11, 12^

## Results

Characteristics of the GMS population over time are included in Table 2. The GMS population decreased from 1,735,534 in 2016 to 1,554,759 in 2020, and increased to 1,581,294 in 2021. The 75+ years age group was the largest group throughout the study period, ranging from 13.0% in 2016 to 15.8% in 2021. Conversely, 0–4 years age group was consistently the smallest, ranging from 5.8% in 2016 to 4.4% in 2021. Females made up the majority of the GMS population throughout the study period, increasing from 53.1% in 2016 to 54.1% in 2021.

**Table 2.** Characteristics of the General Medical Services (GMS) population over time.

	2016	2017	2018	2019	2020	2021
Age group (years) N %						
0–4	100,242 5.78%	88,132 5.34%	82,103 5.16%	77,471 4.96%	74,767 4.81%	68,711 4.35%
5–11	187,675 10.81%	174,060 10.56%	166,760 10.47%	162,127 10.39%	160,585 10.33%	160,806 10.17%
12–15	102,509 5.91%	97,485 5.91%	93,662 5.88%	92,568 5.93%	94,723 6.09%	99,054 6.26%
16–24	168,084 9.68%	153,603 9.31%	145,733 9.15%	134,638 8.63%	125,538 8.07%	135,813 8.59%
25–34	178,306 10.27%	151,514 9.19%	132,226 8.30%	120,891 7.74%	116,980 7.52%	118,626 7.50%
35–44	211,173 12.17%	196,223 11.90%	179,404 11.27%	171,786 10.96%	168,337 10.83%	171,482 10.84%
45–54	189,374 10.91%	186,155 11.29%	180,214 11.32%	183,261 11.74%	184,379 11.86%	186,366 11.79%
55–64	168,190 9.69%	168,288 10.21%	168,530 10.58%	171,786 11.00%	174,236 11.21%	177,423 11.22%
65–69	96,420 5.56%	96,125 5.83%	96,556 6.06%	94,450 6.05%	93,391 6.01%	92,771 5.87%
70–74	108,475 6.25%	111,113 6.74%	114,768 7.21%	116,929 7.49%	119,676 7.70%	120,832 7.64%
75+	225,076 12.97%	226,307 13.72%	232,239 14.59%	235,775 15.10%	242,147 15.57%	249,410 15.77%
Sex N %						
Female	920,815 53.06%	880,329 53.39%	853,493 53.60%	839,738 53.79%	838,767 53.95%	855,759 54.12%
Male	814,709 46.95%	768,676 46.61%	738,702 46.40%	721,279 46.21%	715,992 46.05%	725,545 45.88%
**Total**	1,735,524	1,649,005	1,592,195	1,561,008	1,554,7591	1,581,294

**Figure 1.**
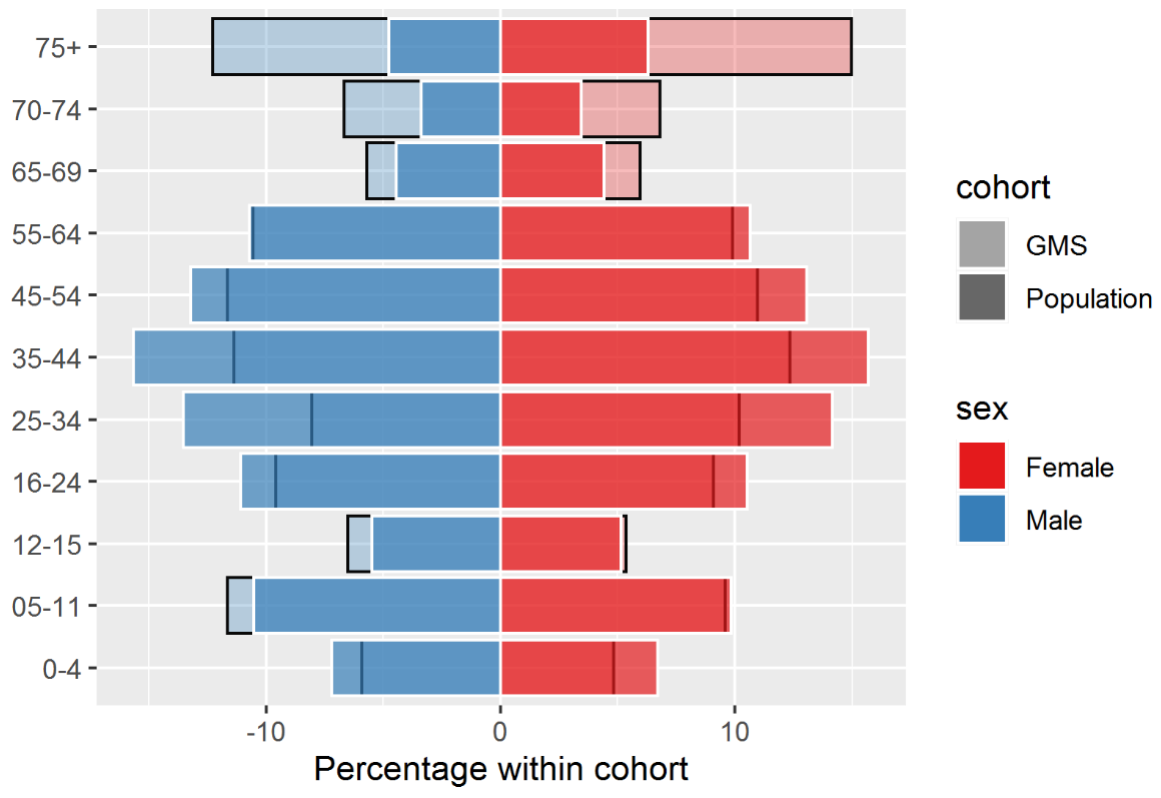
Percentage of males and females in each age group within the General Medical Services (GMS) scheme (light-coloured, black-bordered bars) and full population (bold-coloured, white-bordered bars) in 2016.

**Figure 2.**
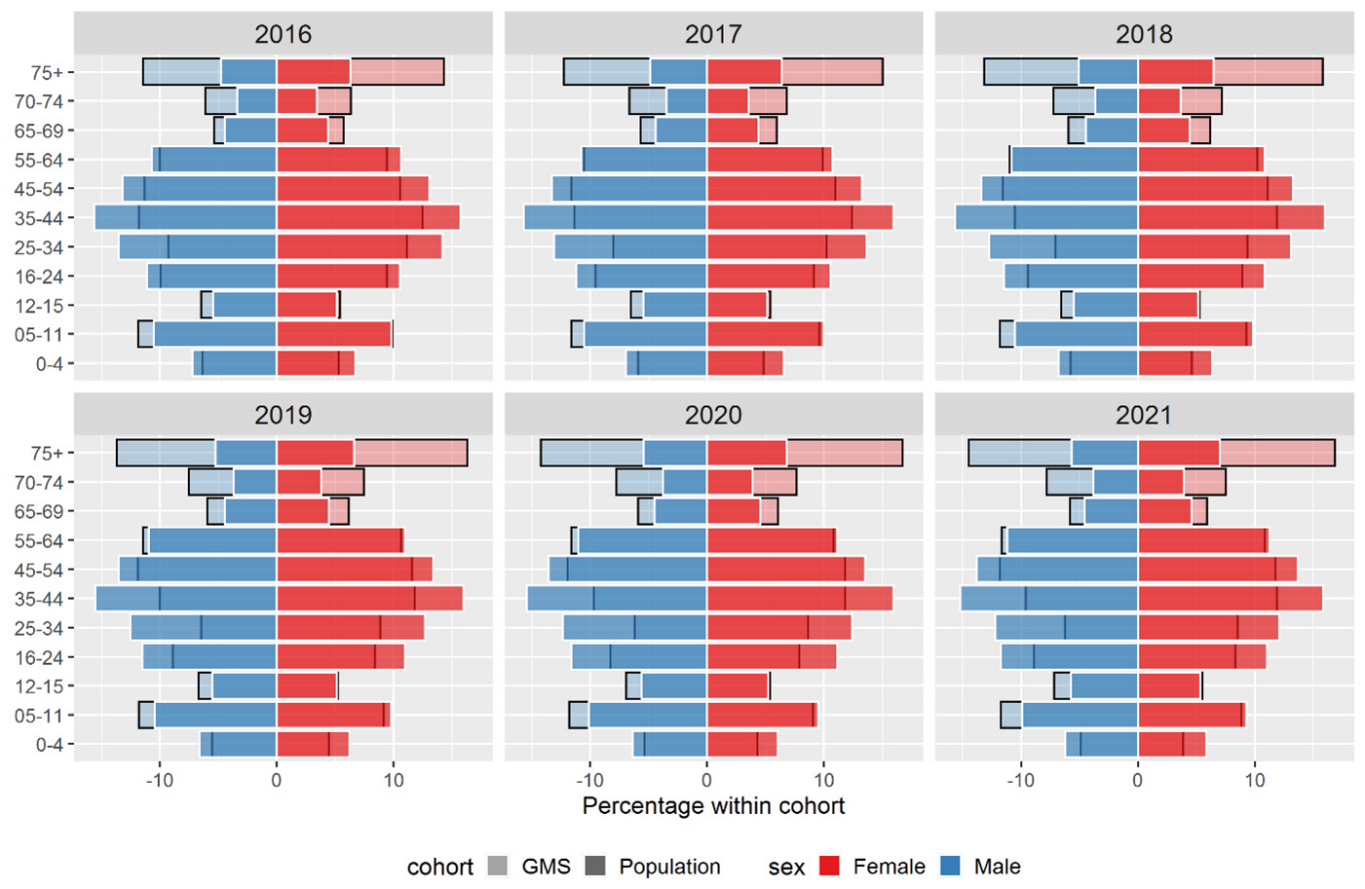
Percentage of males and females in each age group within the General Medical Services (GMS) scheme (light-coloured, black-bordered bars) and full population (bold-coloured, white-bordered bars) for 2016-2021.

Table 3 shows the eligibility rates and relative rates by age group and sex. In the 75+ years age group, 85.2% of the total population were eligible for the GMS scheme in 2016, decreasing to 78.2% in 2021. The age group with the lowest rate of eligible individuals was the 25-34 age group, with 27.0% eligible in 2016 and 19.5% in 2021. The eligibility rate was higher among females compared to males throughout the study period, with 38.2% eligible in 2016 and 33.9% in 2021, compared to 34.6% and 29.2% for males. Figure 1 shows a population pyramid, depicting the percentage of males and females across each age group within GMS eligible individuals, compared to the full population for 2016. The 75+ age group was the most overrepresented in the GMS population in 2016. Among males, individuals aged 75 and over made up approximately 5% of the full population and 12% of the GMS population, while for females, individuals aged 75 and over made up approximately 6% of the full population and 15% of the GMS population. Those aged 5–15 years, and to a greater extent those 65+ years, were overrepresented across all years, and females were overrepresented relative to males (see Figure 2 for population pyramids across years).

**Table 3.** General medical services (GMS) scheme eligibility rate and relative rate over time.

	2016		2017		2018		2019		2020		2021	
	Eligibility rate	Relative rate	Eligibility rate	Relative rate	Eligibility rate	Relative rate	Eligibility rate	Relative rate	Eligibility rate	Relative rate	Eligibility rate	Relative rate
Age group (years)												
0–4	30.2%	0.83	27.2%	0.789	25.7%	0.784	24.5%	0.775	24.2%	0.773	22.7%	0.72
5–11	38.7%	1.063	35.5%	1.031	33.6%	1.026	32.6%	1.029	32.8%	1.05	33.4%	1.06
12–15	40.6%	1.115	38.3%	1.113	36.3%	1.107	35.3%	1.114	35.2%	1.126	35.7%	1.131
16–24	32.6%	0.896	29.4%	0.855	26.9%	0.822	24.3%	0.767	22.2%	0.71	23.8%	0.755
25–34	27.0%	0.742	23.7%	0.688	21.1%	0.643	19.5%	0.615	19.0%	0.608	19.5%	0.618
35–44	28.3%	0.776	25.9%	0.753	23.4%	0.712	22.0%	0.694	21.6%	0.691	22.0%	0.699
45–54	30.2%	0.83	29.3%	0.85	27.8%	0.849	27.7%	0.873	27.3%	0.875	27.1%	0.858
55–64	33.0%	0.907	32.6%	0.947	32.0%	0.977	31.8%	1.004	31.6%	1.011	31.5%	0.999
65–69	45.6%	1.252	45.5%	1.322	44.8%	1.365	43.1%	1.358	41.6%	1.333	40.5%	1.283
70–74	66.8%	1.834	65.7%	1.908	64.6%	1.971	63.3%	1.994	62.7%	2.008	62.2%	1.971
75+	85.2%	2.339	84.0%	2.441	83.0%	2.531	80.7%	2.544	79.4%	2.531	78.2%	2.479
Demographic group												
0–15	36.6%	0.945	33.6%	0.917	31.9%	0.908	30.9%	0.907	30.9%	0.917	31.0%	0.912
16–64	29.9%	0.774	27.9%	0.759	25.9%	0.737	24.8%	0.727	24.1%	0.716	24.6%	0.725
65+	67.4%	1.743	66.7%	1.819	65.9%	1.874	64.2%	1.883	63.2%	1.877	62.4%	1.838
Sex												
Female	38.2%	1.049	36.4%	1.057	34.8%	1.062	33.8%	1.066	33.4%	1.069	33.9%	1.072
Male	34.6%	0.949	32.4%	0.942	30.7%	0.937	29.6%	0.933	29.0%	0.93	29.2%	0.927

Crude and adjusted eligibility rates over time are included in Table 4. The crude rate decreased from 36.4% in 2016 to 31.2% in 2020, with a slight increase to 31.6% in 2021. After directly standardising the rate to the 2016 population based on age group and sex, a similar pattern was observed with 30.8% (95% confidence interval 30.88% to 30.96%) adjusted eligibility rate in 2021.

**Table 4.** General Medical Services (GMS) scheme crude eligibility rate over time, and rate directly standardised to 2017 population based on age group and sex.

	Total estimated population	Total eligible	Crude rate	Standardised rate (95% CI)
**2016**	4,761,865	1,735,524	36.4%	36.4%
**2017**	4,792,490	1,649,005	34.4%	34.29% (34.25-34.33)
**2018**	4,857,015	1,592,195	32.8%	32.50% (32.46-32.54)
**2019**	4,921,496	1,561,008	31.7%	31.28% (31.25-31.32)
**2020**	4,977,443	1,554,759	31.2%	30.66% (30.62-30.70)
**2021**	5,011,460	1,581,294	31.6%	30.80% (30.76-30.84)

**Figure 3.**
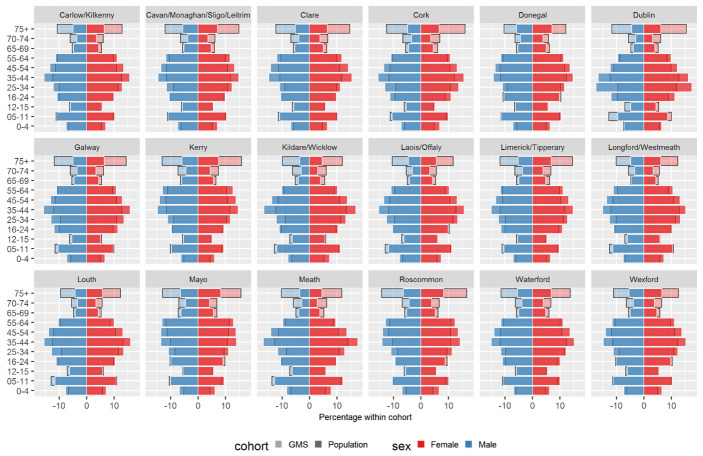
Percentage of males and females in each age group within the GMS scheme (light-coloured, black-bordered bars) and full population (bold-coloured, white-bordered bars) for 2016 across geographic areas.

**Figure 4.**
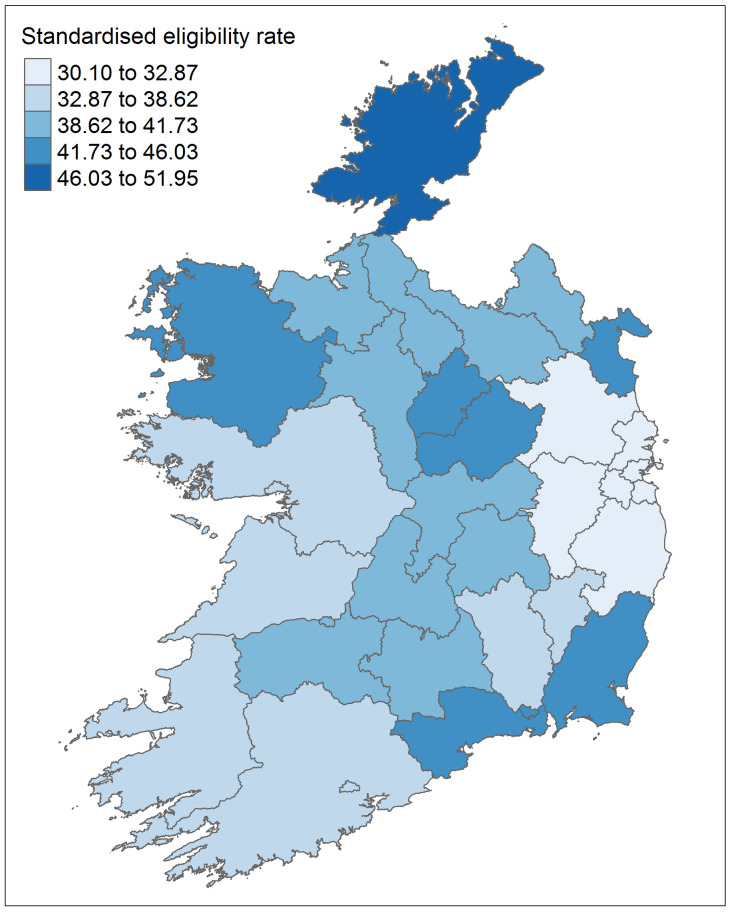
Eligibility rate for the General Medical Services (GMS) scheme by geographical area for 2016, standardised to the national population by age group and sex.

Table 5 shows the crude eligibility rate by area for 2016, and rates directly standardised to the national population based on age group and sex. Population pyramids by area are shown in Figure 3. The highest rate was seen in Donegal, with a crude rate of 52.8% and an adjusted rate of 52.0% (95% CI 51.7% to 52.2%). Dublin had the lowest rate, with a crude rate of 29.3% and an adjusted rate of 30.1% (95% CI 30.0% to 30.2%). Figure 4 shows a map of the adjusted eligibility rates across areas.

**Table 5.** General Medical Services (GMS) scheme crude eligibility rate by area for 2016, and rate directly standardised to national population based on age group and sex.

	Total estimated population	Total eligible	Crude rate	Standardised rate (95% CI)
**Carlow/Kilkenny**	156,164	57,933	37.1%	37.0% (36.8-37.2)
**Cavan/Monaghan/Sligo/Leitrim**	235,141	96,135	40.9%	40.3% (40.1-40.5)
**Clare**	118,817	45,233	38.1%	37.7% (37.4-37.9)
**Cork**	542,868	189,302	34.9%	34.8% (34.6-34.9)
**Donegal**	159,192	84,050	52.8%	52.0% (51.7-52.2)
**Dublin**	1,347,359	394,717	29.3%	30.1% (30.0-30.2)
**Galway**	258,058	95,684	37.1%	37.0% (36.8-37.2)
**Kerry**	147,707	58,482	39.6%	38.6% (38.4-38.9)
**Kildare/Wicklow**	364,929	115,644	31.7%	32.7% (32.5-32.8)
**Laois/Offaly**	162,658	67,396	41.4%	41.7% (41.5-42.0)
**Limerick/Tipperary**	354,452	145,559	41.1%	40.6% (40.4-40.7)
**Longford/Westmeath**	129,643	55,735	43.0%	43.0% (42.7-43.2)
**Louth**	128,884	57,679	44.8%	45.0% (44.7-45.2)
**Mayo**	130,507	60,788	46.6%	45.1% (44.8-45.3)
**Meath**	195,044	61,853	31.7%	32.9% (32.7-33.1)
**Roscommon**	64,544	27,731	43.0%	41.7% (41.3-42.0)
**Waterford**	116,176	54,180	46.6%	46.0% (45.8-46.3)
**Wexford**	149,722	67,423	45.0%	44.5% (44.3-44.8)

## Discussion

The decreasing trend in eligibility rate from 2017 is consistent with previous analysis, which identified a rise from approximately 30% to over 40% from 2008 to 2013 (during the economic crisis affecting Ireland), followed by a decrease in 2014 and 2015 ^13^. The slight increase in eligibility rate from 2020 to 2021 could be partially attributable to the adverse financial impact of the coronavirus disease 2019 (COVID-19) pandemic ^14^.

As expected given that eligibility is often based on income, the areas we identified as having the highest rates are somewhat similar to those with higher levels of deprivation ^15^. However, because of the grouping of areas in our analysis to allow for comparable population and eligibility figures, many of the highest deprivation areas were grouped with others, precluding direct comparison.

Considering representativeness of health data drawn from routine sources (as non-random samples) is important for assessing the external validity of research. A recently published analysis of the OpenSAFELY data, derived from GP records in England and used extensively since the onset of the COVID-19 pandemic to generate evidence, suggests some geographic variation but otherwise good population representativeness ^16^. Even for prospectively collected research data, such as the UK BioBank, consideration of representativeness and the potential for healthy user bias is important ^17^. As well as undermining generalisability, selection bias may also impact internal validity, where sampling into the study is affected by both the exposure and outcome of interest, and thus the exposure-outcome association may be biased ^18^. As the GMS scheme is means tested, older people and individuals from lower socioeconomic backgrounds are overrepresented, which may have implications for research using this data and the conclusions drawn from it.

### Limitations

For the year-by-year analysis, the population figures for 2017–2021 are estimated based on projections from the census, which may not fully capture true population changes. The next census was conducted in April 2022 (delayed from April 2021 due to the COVID-19 pandemic). The publication of data from this (expected to be released in stages between 2023 and 2024 ^19^) will provide an opportunity to evaluate contemporary eligibility rate using actual population figures, and to evaluate how eligibility patterns have changed by area over time.

Our area-level analysis required grouping of several counties and included only 18 areas, compared to 32 LHOs and 31 local authority areas. All of Dublin’s local authority areas were grouped into one area, resulting in an area with a very high population and no possibility of within area comparisons. As mentioned above, this meant that a closer examination of deprived and affluent areas was not possible. Releasing data at a more granular geographic area, or providing information to map townland-level Census figures to LHO areas for example, would allow for a more detailed analysis. In general alignment and harmonisation of how data is presented by various statutory bodies in health and elsewhere would enhance the secondary use of such data for research and other purposes.

## Conclusion

This study uses the most up-to-date data available to provide age group, sex and area-based figures for GMS eligibility which may inform planning and conduct of research focusing on GMS-eligible individuals. We also provide the statistical code to import open data (where available) and conduct analysis, along with extracted data from the PCRS portal. Provision of open, interoperable PCRS eligibility data per month via Ireland’s Open Data Portal would enhance the usability of this data for research and wider purposes.

## Data Availability

Zenodo: Data and code related to “Eligibility rates and representativeness of the General Medical Services scheme population in Ireland 2017–2021: A methodological report.” https://doi.org/10.5281/zenodo.7097254 ^9^. This project contains the following underlying data: age_sex_lho_eligibility_2016.xlsx (GMS eligibility figures by age group, sex, and LHO for 2016) age_sex_lho_eligibility_2017-2021.xlsx (GMS eligibility figures by age group, sex, and LHO for 2017–2021) Additionally, data from the CSO datasets E3003 (Population 2011 to 2016) and PEA11 (Population estimates from 1926) were downloaded from the CSO website as described in the Stata do-file included in the repository. Data are available under the terms of the Creative Commons Attribution 4.0 International license (CC-BY 4.0).
