# Supplementary material for: Eligibility rates and representativeness of the General Medical Services scheme population in Ireland 2016-2021: A methodological report
Source: HRB Open Res. 2023 Oct 18;5:67. Originally published 2022 Oct 20. [Version 2] doi: 10.12688/hrbopenres.13622.2 (PMC10518849; doi:10.12688/hrbopenres.13622.2)
Supplement: Peer review history

# Reviewer report: Reviewer response for version 1

**DOI:** 10.21956/hrbopenres.14894.r35745

**Published:** 2023-09-13

**Authors:** Geoffrey M. Curran

**Affiliations:** 1 Department of Pharmacy Practice, University of Arkansas for Medical Sciences, Little Rock, AR, USA

I found the article to be a relatively straightforward presentation of potential trends in eligibility for the Irish GMS scheme. As an outsider to the Irish health system, it helped me to better understand the GMS scheme and to appreciate geographic differences in Ireland in terms of economic outlook and resultant eligibility. I appreciated the transparency of the authors and their support of others gaining access to the same data for their own analysis.

 There were several areas where I would have liked more description and/or discussion. I would have appreciated more information on the basis of (or assumptions applied for) the projections for the "out years." (I was not sure what the authors meant by "rated were directly standardized" to the 2016 population). I also could have used some more information on the possible economic or other social forces that could have been driving the projected changes in eligibility and geographic differences. Also, I am not sure that the readers were given a reason why they were required to group several counties. I also could have used a brief discussion of how specifically these data could be used in the "planning and conduct of research focusing on GMS-eligible individuals."

 In general, I found the tables followable and useful. I struggled some with the figures. It took me a while see and understand the white/black bar distinction.  

 I agree with the feedback provided by the first reviewer. I appreciated the opportunity to review the manuscript. I had not seen previously a system whereby a review is sought after publication.

Is the work clearly and accurately presented and does it cite the current literature?

Yes

If applicable, is the statistical analysis and its interpretation appropriate?

I cannot comment. A qualified statistician is required.

Are all the source data underlying the results available to ensure full reproducibility?

Yes

Is the study design appropriate and is the work technically sound?

Yes

Are the conclusions drawn adequately supported by the results?

Yes

Are sufficient details of methods and analysis provided to allow replication by others?

Yes

Reviewer Expertise:

Health services research, implementation science

I confirm that I have read this submission and believe that I have an appropriate level of expertise to confirm that it is of an acceptable scientific standard.

# Reviewer report: Reviewer response for version 1

**DOI:** 10.21956/hrbopenres.14894.r33160

**Published:** 2022-11-22

**Authors:** Amelia Smith

**Affiliations:** 1 Medicines Management Programme, Health Service Executive, Trinity Centre for Health Sciences, St. James’ Hospital, Dublin, Ireland; 2 Department of Pharmacology and Therapeutics, Trinity College Dublin, Trinity Centre for Health Sciences, St. James’ Hospital, Dublin, Ireland

Well done to the authors on a well-written and informative study. 

 Introduction:

1. Final paragraph; include sentence to explain that there’s very limited information on healthcare use, dispensed claims, etc. in the privately funded cohort.
2. The authors could include a brief description of the Drugs Payment Scheme (DPS) as all persons not eligible for GMS are eligible for DPS. This could sit in the discussion, either.

Results:

1. ‘The GMS population decreased from 1,735,534 in 2016 to 1,554,759 in 2020, and increased to 1,581,294 in 2021.’ Double check figures in Table 2 – states the 2016 as 1,735,524.
2. Check total figures for 2019 and 2020 in Table 2.
3. For the population pyramids, the grey legend for cohort (GMS or Population) isn’t intuitive. I think this could be removed/changed, as the Figure description is more informative.
4. Page 4, 2 ^nd^ column, 3 ^rd^ paragraph, final sentence: ‘(95% confidence interval 30.88% to 30.96%)’ these CI’s are different to that in Table 4.

Discussion:

1. Authors could include a brief discussion on the practical implications of the difference in eligibility rates across age groups – the aging population and fiscal implications going forward, etc. However, this is just a suggestion – not a requirement.

Is the work clearly and accurately presented and does it cite the current literature?

Yes

If applicable, is the statistical analysis and its interpretation appropriate?

Yes

Are all the source data underlying the results available to ensure full reproducibility?

Yes

Is the study design appropriate and is the work technically sound?

Yes

Are the conclusions drawn adequately supported by the results?

Yes

Are sufficient details of methods and analysis provided to allow replication by others?

Yes

Reviewer Expertise:

Drug utilisation studies using PCRS pharmacy claims data. Health technology management.

I confirm that I have read this submission and believe that I have an appropriate level of expertise to confirm that it is of an acceptable scientific standard.
